# Glial fibrillary acidic protein in cerebrospinal fluid of patients with spinal muscular atrophy

**DOI:** 10.1002/acn3.51645

**Published:** 2022-08-11

**Authors:** Maren Freigang, Petra Steinacker, Claudia D. Wurster, Olivia Schreiber‐Katz, Alma Osmanovic, Susanne Petri, Jan C. Koch, Kevin Rostásy, André Huss, Hayrettin Tumani, Benedikt Winter, Björn Falkenburger, Albert C. Ludolph, Markus Otto, Andreas Hermann, René Günther

**Affiliations:** ^1^ Department of Neurology University Hospital Carl Gustav Carus, Technische Universität Dresden Dresden Germany; ^2^ Department of Neurology Universitätsklinikum Halle (Saale) Halle (Saale) Germany; ^3^ Department of Neurology Ulm University Ulm Germany; ^4^ Department of Neurology Hannover Medical School Hannover Germany; ^5^ Essener Zentrum für Seltene Erkrankungen (EZSE) University Hospital Essen Essen Germany; ^6^ Department of Neurology University Medicine Göttingen Göttingen Germany; ^7^ Department of Pediatric Neurology Children's Hospital Datteln, University Witten/Herdecke Datteln Germany; ^8^ Deutsches Zentrum für Neurodegenerative Erkrankungen (DZNE) Ulm Ulm Germany; ^9^ Department of Pediatric Neurology University Hospital Mannheim Mannheim Germany; ^10^ Deutsches Zentrum für Neurodegenerative Erkrankungen (DZNE) Dresden Dresden Germany; ^11^ Translational Neurodegeneration Section “Albrecht‐Kossel”, Department of Neurology, and Center for Transdisciplinary Neurosciences Rostock (CTNR) University Medical Center Rostock, University of Rostock Rostock Germany; ^12^ Deutsches Zentrum für Neurodegenerative Erkrankungen (DZNE) Rostock/Greifswald Rostock Germany

## Abstract

**Objective:**

Activated astroglia is involved in the pathophysiology of neurodegenerative diseases and has also been described in animal models of spinal muscular atrophy (SMA). Given the urgent need of biomarkers for treatment monitoring of new RNA‐modifying and gene replacement therapies in SMA, we examined glial fibrillary acidic protein concentrations in cerebrospinal fluid (cGFAP) as a marker of astrogliosis in SMA.

**Methods:**

58 adult patients and 21 children with genetically confirmed 5q‐associated SMA from four German motor neuron disease specialist care centers and 30 age‐ and sex‐matched controls were prospectively included in this study. cGFAP was measured and correlated to motor performance and disease severity. Additionally, we compared cGFAP with neurofilament light chain concentrations in cerebrospinal fluid (cNfL).

**Results:**

cGFAP concentrations did not differ from controls but showed higher levels in more severely affected patients after adjustment for patients' age. Normalized cNfL values were associated with disease severity. Within 14 months of nusinersen treatment, cGFAP concentrations did not change, while cNfL decreased significantly.

**Interpretation:**

cGFAP is not an outstanding biomarker in SMA, but might support the hypothesis that glial activation is involved in SMA pathology. Unlike previously suggested, cNfL may be a promising biomarker also in adult patients with SMA, which should be subject to further investigations.

## Introduction

5q‐associated spinal muscular atrophy (SMA) is a lower motor neuron disease based on a lack of survival of motor neuron (SMN) protein caused by a loss‐of‐function mutation of the *Survival of motor neuron 1* gene *(SMN1)*.^1^ The deficiency of SMN protein primarily leads to death of motor neurons. However, selective depletion of SMN protein within motor neurons of mice only generates a very mild, late‐onset SMA‐like phenotype,^2^ which contrasts the severe phenotype resulting from ubiquitously low expression of SMN protein.^3^ These findings suggest that SMA pathophysiology is more complex and involves non‐neuronal tissue (e.g. astrocytes) apart from motor neurons alone.^2^ Astrocytes are crucial for neuronal homeostasis, trophic support of neurons and synaptic plasticity. Thus, the involvement of astrocytes is widely accepted in other motor neuron diseases like amyotrophic lateral sclerosis.^4^, ^5^ Glial fibrillary acidic protein (GFAP) is the principal intermediate filament in mature astrocytes, a key element of their cytoskeleton and increased expression of GFAP is an indication of reactive gliosis, a process which has been shown to be highly related to brain damage and aging.^6^ Elevated GFAP concentrations in tissue, serum or cerebrospinal fluid (CSF) already have been reported for several neurodegenerative and neuroinflammatory diseases, stroke, and traumatic brain injuries^6^, ^7^, ^8^, ^9^, ^10^, ^11^, ^12^ and were found to have prognostic as well as predictive value. Astrogliosis, visualized by GFAP staining, has been described in the spinal cord of SMA mice (SMNΔ7) and patients with SMA, especially located in the gray matter of the ventral horn and astrocytic processes form glial bundles along the ventral roots.^3^, ^13^, ^14^, ^15^, ^16^ Likewise, recent research has increasingly focused on the involvement of glial cells in the development and maintenance of SMA and on inferring new strategies for therapeutic approaches.^17^, ^18^ Astrocytes were found to be restructured and showed upregulated GFAP expression as a sign of reactive state prior to the affection of motor neurons in SMA disease.^19^, ^20^ In SMA models, viral‐based restoration of SMN protein levels in astrocytes attenuated disease progression and improved neuromuscular integrity.^3^ Astrocytes, derived from induced pluripotent stem cells from SMA mice, showed morphological and functional changes as signs of activation and astrocyte‐motor neuron co‐cultures presented impaired synaptic formation and interaction.^21^

The aim of this study was to evaluate GFAP concentration in CSF (cGFAP) as a biomarker for disease severity and treatment monitoring in patients with SMA and to further investigate the non‐neuronal involvement in the pathophysiology of SMA. Furthermore, we aimed to compare cGFAP with neurofilament light chain (cNfL) as a marker of neurodegeneration and with chitotriosidase 1 (cCHIT1) as a marker of neuroinflammation in CSF. cCHIT1 has recently been shown to be elevated in treatment‐naïve patients with SMA.^22^ cNfL has been found to reflect disease severity and treatment response in pediatric SMA,^23^ but still provides limited information in adult SMA because it overlaps with healthy controls and often shows no association with motor improvement.^24^, ^25^, ^26^, ^27^

## Methods

### Standard protocol approvals, registrations, and patient consents

58 adult patients and 21 children with genetically confirmed 5q‐associated SMA from 4 German motor neuron disease specialist care centers (Departments of Neurology in Dresden, Ulm, Hannover and Göttingen) and 30 age‐ and sex‐matched controls were prospectively included in this study between 2017 and 2020. The local ethics committees of all participating sites approved the study and all patients signed written informed consent.

The demographic and clinical data of patients were collected including age, sex, disease onset, baseline weight and height, clinical subtype, number of *SMN2* copies if available and ambulatory status.

Patients received nusinersen treatment according to the prescribing information for up to 14 months (Visit 7 = V7). CSF was obtained by lumbar puncture (LP), which was performed for intrathecal administration of nusinersen.

The samples designated for GFAP assay were stored at −80°C within 2 h after centrifugation (5 min; 800*g*; RT). In total, 214 CSF samples were analyzed for GFAP concentration at three time points (V1 = baseline, V5 = 6.2 ± 0.6 months, V7 = 14.2 ± 0.9 months) using ELISA kits (BioVendor, Brno, Czech Republic) at 1:3 dilution according to the instructions of the manufacturer. For quality control, a CSF pool was measured in triplicates per plate additionally to duplicates of the control samples included in the GFAP ELISA kits. The mean intra‐assay and inter‐assay coefficients of variation were <15% for both the kit controls and the CSF pool. One patient was excluded from the analysis since the CSF sample of that patient was insufficient for GFAP determination at baseline. cNfL levels were determined by the ELLA microfluidic system (Bio‐Techne, Minneapolis, USA) and measurements were performed according to the manufactures instructions. In more detail, CSF samples were diluted 1:4 and analyzed in technical triplicates with an acceptance threshold of 10% coefficients of variation. In addition, chitotriosidase 1 concentrations in CSF (cCHIT1) were measured in the same cohort and at the same time as cGFAP (previously published in Ref. [22]).

To monitor motor and functional outcome, established motor scores (Hammersmith Functional Motor Scale Expanded—HFMSE,^28^ Revised Upper Limb Module—RULM,^29^ Children's Hospital of Philadelphia Infant Test of Neuromuscular Disorders—CHOP INTEND^30^) as well as the revised ALS‐Functional Rating Scale (ALSFRS‐R)^31^ were assessed concurrently at each visit. Motor scores comprise several items rating different motor skills with higher scores indicating better function. Ratings were performed according to the respective manuals.

### Statistical analysis

Statistical analysis and data visualization were performed using SPSS Statistics 27 (IBM, Chicago (IL), USA) and GraphPad Prism 5 (GraphPad Software Inc., San Diego (CA), USA). Unless otherwise stated, cGFAP and cNfL data and the assessed scores are presented as median ± interquartile range (IQR). cGFAP and cNfL data were not normally distributed as tested by Shapiro–Wilk test (*p* < 0.001). We therefore applied rank‐based, non‐parametric tests for the baseline analysis. To estimate the comparability of study group and control group, we used Pearson's Chi‐squared test for equal distribution regarding sex and Mann–Whitney U test concerning conformity of age. To investigate the association between cGFAP values and disease severity, we correlated cGFAP baseline values with demographic features and clinical assessments using Spearman's rank correlation coefficient (*ρ*). Due to the significant association with age, we considered it a confounding factor and controlled for baseline age by partial correlation regarding both cGFAP and cNfL. A correlation coefficient of *ρ* < 0.3 was considered as a weak, *ρ* = 0.3–0.59 as a moderate, and *ρ* > 0.6 as a strong correlation (modified from^32^). We used Mann–Whitney *U* test or one‐way analysis of covariance (ANCOVA) with post‐hoc Bonferroni adjustment for comparison of cGFAP or cNfL (dependent variables) between different patient subgroups considering age as covariate. To meet the assumptions of ANCOVA, we subjected the cGFAP data to a square root transformation and the cNfL data to an inverse and log transformation. For longitudinal analysis under nusinersen treatment, we performed Wilcoxon signed‐rank test to include all available data (*N* = 58) for the comparison between baseline (V1) and 14‐month follow‐up (V7, representing third maintenance dose). Data sets with missing values were excluded pairwise for cross‐sectional and longitudinal analysis. Statistical significance threshold was set to <0.05.

## Results

**Figure 1:**
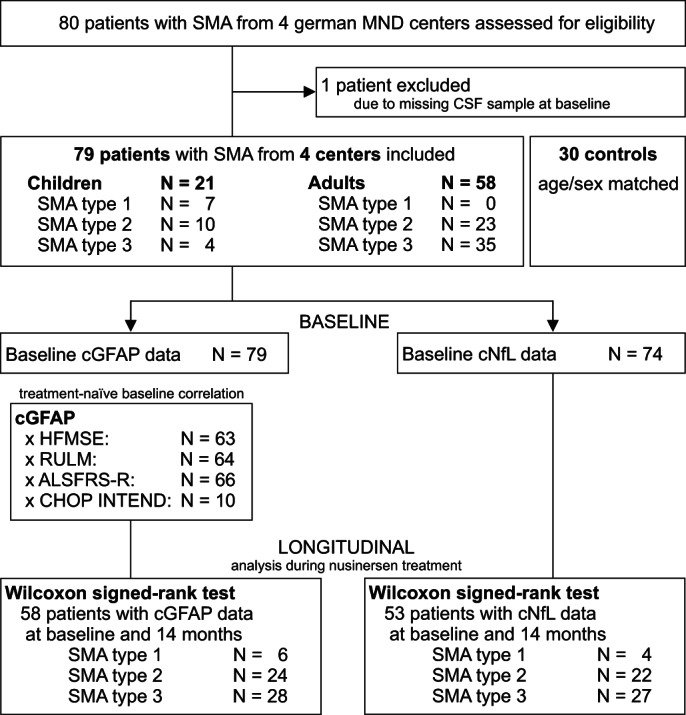
Study profile. SMA, spinal muscular atrophy; MND, motor neuron disease; cGFAP, glial fibrillary acidic protein concentration in cerebrospinal fluid; cNfL, neurofilament light chain concentration in cerebrospinal fluid; HFMSE, Hammersmith Functional Motor Scale Expanded; RULM, Revised Upper Limb Module; ALSFRS‐R, revised ALS Functional Rating Scale; CHOP INTEND, Children's Hospital of Philadelphia Infant Test of Neuromuscular Disorders.

58 adult patients and 21 children with SMA type 1 (*N* = 7), type 2 (*N* = 33) or type 3 (*N* = 39) were included in the analysis. Median age was 31 years (IQR 17–43), 52% were female. The control group was age‐ and sex‐matched and comprised 23 adults and 7 children without neurodegenerative or neuroinflammatory disease (healthy controls: *N* = 23, normal pressure hydrocephalus: *N* = 3, idiopathic Bell's palsy: *N* = 4). In the control group, median age was 30 years (IQR 17–44), 60% were female. The distribution of sex or age did not differ significantly between the groups. Details of study group characteristics and study profile are presented in Table 1 and Figure 1.

**Table 1:** Study group characteristics.

	SMA (*N* = 79)	Controls (*N* = 30)
Age [year], median (IQR)	31 (17–43)	30 (17–44)
Age of onset [year], median (IQR)	1 (0–3)	
Disease duration [year], median (IQR)	28 (15–37)	
Sex, *N* (%)		
Female	41 (52)	18 (60)
Male	38 (48)	12 (40)
SMA type, *N* (%)		
1	7 (9)	
2	33 (42)	
3	39 (49)	
*SMN2* copy number, *N* (%)		
2	9 (16)	
3	31 (53)	
4+	18 (31)	
Unknown	21	
Weight [kg], median (IQR)	50 (33–65)	
Height [cm], median (IQR)	158 (145–170)	
BMI [kg/m^2^], median (IQR)	20.5 (16.1–23.4)	
Scoliosis, *N* (%)		
Present	50 (63)	
Not present	29 (37)	
Spondylodesis, *N* (%)		
Present	24 (30)	
Not present	55 (70)	
Wheelchair‐use, *N* (%)		
Never	9 (11)	
Occasionally	6 (8)	
Permanently	64 (81)	
Mobility, *N* (%)		
Never able to walk	40 (51)	
Lost ability to walk	24 (30)	
Still able to walk	15 (19)	

IQR, interquartile range; BMI, body mass index; *SMN2*, *Survival of motor neuron 2* gene.

### cGFAP and cNfL concentrations in treatment‐naïve patients were not elevated, but indicated disease severity

**Figure 2:**
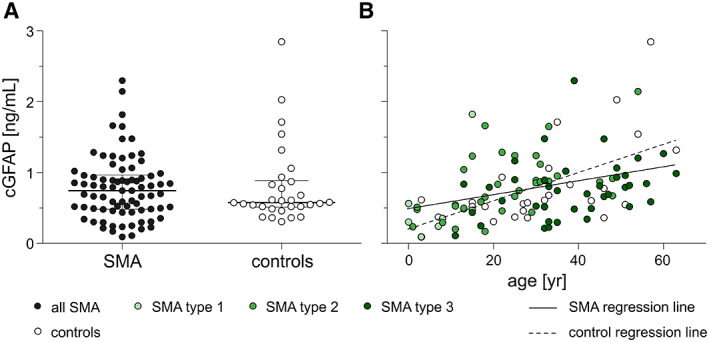
cGFAP concentrations before treatment initiation. (A) Baseline cGFAP concentrations comparing diseased individuals (closed circles; *N* = 79) to controls (open circles; *N* = 30). Horizontal line shows median, whiskers illustrate interquartile range (0.25–0.75), each icon represents an individual patient. (B) Correlation between age and cGFAP concentration before treatment initiation; each icon represents an individual person; shades of green distinguish SMA type, open circles display controls. Solid line shows regression line of patients with SMA, dashed line shows regression line of controls. cGFAP, glial fibrillary acidic protein concentration in cerebrospinal fluid. [Colour figure can be viewed at wileyonlinelibrary.com]

cGFAP concentrations of treatment‐naïve patients with SMA did not differ from the control group (*F* (1, 105) = 0.024, *p* = 0.877; Fig. 2A, Table 2). cGFAP concentrations correlated with patients' (*ρ* = 0.405, *p* < 0.001, *N* = 79; Fig. 2B) and controls' age (*ρ* = 0.544, *p* = 0.002, *N* = 30; Fig. 2B), but were not associated with age of disease onset, height, weight or *SMN2* copy number (see Table 3). Higher cGFAP levels were associated with lower motor function (Table 4) in treatment‐naïve patients. Furthermore, cGFAP concentrations did not correlate with cCHIT1 concentrations which was measured within the same cohort at the same time (*ρ* = 0.081, *p* = 0.483, *N* = 79; calculated by partial rank correlation controlling for patients' age and height; Figure S1 displays scatter dot plot of raw data).^22^ cGFAP concentrations were higher in patients with SMA type 2 compared to type 3 (*F* (2, 74) = 3.673, *p* < 0.05, partial *η* ^2^ = 0.090) after adjustment for patients' age, but did not differ between SMA type 1 versus 2 or type 1 versus 3 or compared to controls or regarding *SMN2* copy number (*F* (4, 51) = 0.333, *p* = 0.855). Moreover, patients who were able to walk had lower cGFAP concentrations than patients who were not (*F* (1, 75) = 4.813, *p* < 0.05, partial *η* ^2^ = 0.060).

**Table 2:** cGFAP levels in treatment‐naïve patients with SMA and controls.

	SMA (*N* = 79)	Controls (*N* = 30)
cGFAP [ng/mL],		
median	0.743	0.578
(IQR)	(0.479–0.959)	(0.512–0.887)
range	0.091–2.295	0.305–2.843

cGFAP, glial fibrillary acidic protein concentration in cerebrospinal fluid; IQR, interquartile range.

**Table 3:** Correlation between cGFAP concentration and characteristics of treatment‐naïve patients with SMA.

Spearman rho	cGFAP [ng/mL]
Age [year]	*ρ* = 0.405 ***p* < 0.001** *N* = 79
Partial correlation controlled for age	
Height [cm]	*ρ* = 0.050 *p* = 0.663 *N* = 79
Weight [kg]	*ρ* = 0.070 *p* = 0.543 *N* = 79
*SMN2* copy number	*ρ* = −0.080 *p* = 0.554 *N* = 58
Disease onset [year]	*ρ* = −0.216 *p* = 0.061 *N* = 77
Disease duration [year]	*ρ* = 0.173 *p* = 0.136 *N* = 77

cGFAP, glial fibrillary acidic protein concentrationin cerebrospinal fluid; *ρ*, (partial) rank correlation coefficient, significant values are marked in bold.

**Table 4:** Correlation between cGFAP concentration and disease severity scores in treatment‐naïve patients with SMA.

Controlled for age	cGFAP [ng/mL]
HFMSE	*ρ* = −0.381 *p* = **0.002** *N* = 63
RULM	*ρ* = −0.294 *p* = **0.019** *N* = 64
ALSFRS‐R	*ρ* = −0.330 *p* = **0.007** *N* = 66
CHOP INTEND	*ρ* = −0.193 *p* = 0.619 *N* = 10

cGFAP, glial fibrillary acidic protein concentrationin cerebrospinal fluid; HFMSE, Hammersmith Functional Motor Scale Expanded; RULM, Revised Upper Limb Module; ALSFRS‐R, revised ALS Functional Rating Scale; CHOP INTEND, Children's Hospital of Philadelphia Infant Test of Neuromuscular Disorders; *ρ*, partial rank correlation coefficient, significant values are marked in bold.

**Figure 3:**
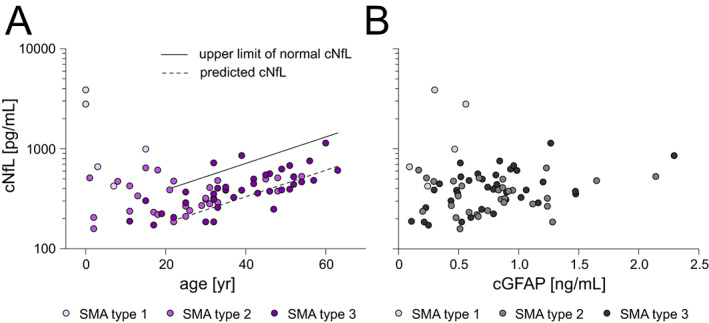
cNfL concentrations before treatment initiation. (A) Association between age and cNfL concentration before treatment initiation; each icon represents an individual person; shades of purple distinguish SMA type. Solid line indicates upper limit of normal (reported by Yilmaz et al.), dashed line shows predicted cNfL concentration (calculated using regression formula determined by Yilmaz et al.). (B) Correlation between cGFAP and cNfL concentration before treatment initiation; each icon represents an individual person; shades of gray distinguish SMA type. cNfL, neurofilament light chain concentration in cerebrospinal fluid; cGFAP, glial fibrillary acidic protein concentration in cerebrospinal fluid; *N* = 73. [Colour figure can be viewed at wileyonlinelibrary.com]

Data for cNfL concentration at baseline was available for 74 patients, because the amount of CSF of five patients was not sufficient for NfL determination. One adolescent patient with SMA type 1 showed a cNfL level of 18272 pg/mL, which was outside of 1.5 fold IQR from the median within the subgroup of SMA type 1 (>5193.75 pg/mL) and has been therefore excluded from the analysis. Finally, the baseline data set represented 73 treatment‐naïve patients with SMA, of whom 20 were younger than 20 years. cNfL levels (for baseline data see Table S1) were within the reference range determined by Yilmaz et al.^33^ in 94% (50/53) of the patients aged 21 to 63 years (Fig. 3A). In contrast, most children with SMA aged 0 to 7 years had clearly increased cNfL levels compared to the mean value of the control individuals reported by Olsson et al.^23^; especially those with SMA type 1 (4/4 above mean + 2SD). cNfL concentration was associated with patients' age (*ρ* = 0.316, *p* < 0.01; *N* = 73). cGFAP and cNfL did not correlate at baseline after adjustment for patients' age (*ρ* = −0.053, *p* = 0.657; df = 70).

In a subset of patients with SMA type 2 (*N* = 24) and 3 (*N* = 34), determination of serum creatinine concentration (sCrn) was done as part of the clinical routine. Patients lacking baseline cGFAP, cNfL, and sCrn were excluded listwise from this subanalysis. In this subset, there was a slight difference between SMA type 2 and 3 for cGFAP (*F* (1, 55) = 4.991, *p* < 0.05, partial *η* ^2^ = 0.083) and no difference regarding cNfL (*p* = 0.532; Fig. S4A). sCrn was higher in patients with SMA type 3 (median sCrn SMA type 2: 11.0 μmol/mL, SMA type 3: 24.5 μmol/mL; *U* = 127.5, z = −4.437, *p* < 0.0001; Fig. S4B), which reflects more preserved muscle mass and suggests more remaining motor units. Higher sCrn‐normalized cGFAP (cGFAP/sCrn: *F* (1, 55) = 36.837, *p* < 0.0001, partial *η* ^2^ = 0.401) and higher sCrn‐normalized cNFL (cNfL/sCrn *F*(1, 55) = 27.311, *p* < 0.0001, partial *η* ^2^ = 0.332) values were found in SMA type 2 compared to SMA type 3 (Fig. S4C). Age was considered as a covariate, since it differed between the subgroups (median age SMA type 2: 28.0 years, SMA type 3: 40.5 years, *U* = 186.5, *z* = −3.500, *p* < 0.001; Fig. S4B).

### cGFAP and cNfL concentrations decreased in patients with SMA following nusinersen treatment

**Figure 4:**
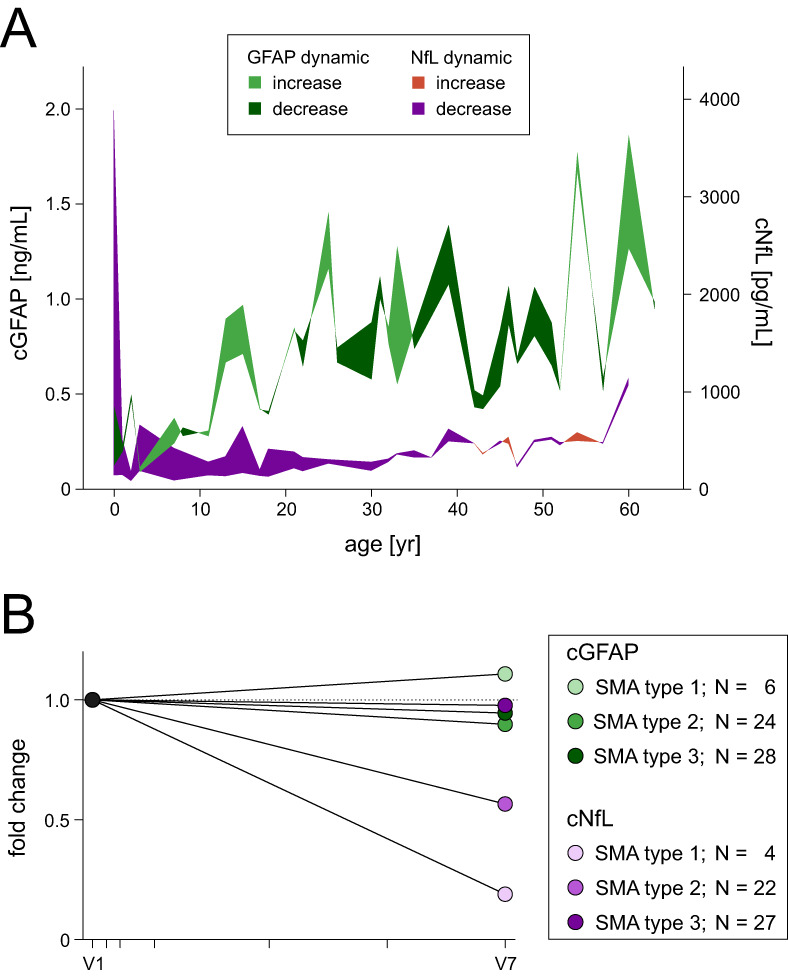
Dynamic of cGFAP and cNfL during nusinersen treatment. (A) Change of cGFAP and cNfL concentration during 14 months of nusinersen treatment plotted against age at start of treatment. Colored areas are created by the difference between baseline and follow‐up for cGFAP and cNfL, respectively. When more than one paired data set was available for the corresponding age, the mean was plotted. Dark green or dark purple areas illustrate a decrease in cGFAP or cNfL, respectively, from baseline to 14‐month follow‐up. (B) Fold change of cGFAP concentrations during nusinersen treatment from baseline (V1) to 14‐month follow‐up (V7). Each colored circle represents the median value of the respective subgroup at follow‐up measurement; color shading distinguishes SMA type. Each tick on the x‐axis indicates a nusinersen administration. cGFAP, glial fibrillary acidic protein concentration in cerebrospinal fluid; cNfL, neurofilament light chain concentration in cerebrospinal fluid. [Colour figure can be viewed at wileyonlinelibrary.com]

After 14 months of nusinersen treatment, cGFAP concentrations did not differ significantly from baseline levels (z = −1.411, *p* = 0.158; Table 5; raw data displayed in Fig. S2). However, lower cGFAP concentrations were observed in two thirds of individuals compared to baseline and the decrease was significant in patients with motor improvement as indicated by increased HFMSE scores after 14 months of nusinersen treatment (median change −11.6%, *z* = −2.374, *p* < 0.05; Table 5). In order to compare our results with those of Olsson et al.,^23^ we screened our study cohort for patients who met the inclusion criteria of Olsson et al. We identified two children with SMA type 1 carrying 2 *SMN2* copies who were treated within their first year of life (#57, #74). Similar to Olsson et al., cGFAP concentrations declined during nusinersen treatment. In fact, cGFAP concentrations decreased by 70% in these two patients with SMA type 1 with recent onset and notably, cNfL concentration decreased and CHOP INTEND scores improved coincidently (Fig. S3). Overall, cNfL decreased in 77.4% (43/53) with a greater decrease in younger patients (*ρ* = 0.626, *p* < 0.001, *N* = 53) and more severely affected patients. Median change was −71 pg/mL (*z* = −4.795, *p* < 0.0001; for details see Table S2 and Fig. 4). In the subset, cGFAP/sCrn ratio did not change during 14 months (*z* = −0.413, *p* = 0.679, *N* = 38) while cNfL/sCrn ratio did overall (*z* = −3.488, *p* < 0.001, *N* = 38) and in SMA type 2 (*z* = −2.675, *p* < 0.01, *N* = 17). The change within SMA type 3 slightly missed significance (*z* = −1.860, *p* = 0.063, *N* = 21; Fig. S4D).

**Table 5:** Dynamics in cGFAP concentration during 14 months of nusinersen treatment.

	14‐month analysis				
	*N*	Median (IQR)	Difference versus baseline		*p* value
			Median (IQR)	(%)	
cGFAP [ng/mL]	58	0.5838 (0.4144–0.8982)	−0.0488 (−0.1199–0.1049)	−7.4	0.158
HFMSE increase	21	0.4725 (0.4104–0.7337)	−0.0700 (−0.2275 to −0.0017)	−11.6	**0.018**
HFMSE decrease	6	0.8858 (0.7337–1.2268)	−0.0371 (−0.2468 to 0.5370)	−4.0	0.753

cGFAP, glial fibrillary acidic protein concentration in cerebrospinal fluid; HFMSE, Hammersmith Functional Motor Scale Expanded; IQR, interquartile range; *p* value calculated by Wilcoxon signed‐rank test, significant values are marked in bold.

## Discussion

After initially assuming that the SMA phenotype was based on a motor neuron‐specific pathology, recent research elucidated a more systemic disorder.^17^, ^34^ Astrocytes play a dual role in this non‐neuronal involvement. As in many other neurodegenerative diseases, reactive astrogliosis was reported in the spinal cord of patients with SMA type 2^16^ and 3^15^ including glial bundles in anterior roots^13^ (for review Ref. [6, 17]). Additionally, astrocytes are crucial in the non‐cell‐autonomous pathophysiology of motor neuron diseases. For SMA, Rindt et al. revealed the importance of astrocytes in SMA pathology since they observed improved life span and motor function after restoration of SMN protein levels specifically in astrocytes.^3^ Interestingly, when restoring SMN protein levels in motor neurons only, improvements were only minor,^35^, ^36^ underlining the importance of the non‐cell‐autonomous effects. Thus, astrocytes might not only be activated secondarily to form a reactive gliosis, for example as a consequence of motor neuron degeneration or triggered by activated microglia, but also might be induced intrinsically by the SMN protein deficiency itself. Since, on the one hand, nusinersen treatment prevents motor neuron degeneration and consequently formation of astrogliosis and on the other hand, nusinersen treatment might restore SMN protein levels also directly in non‐neuronal tissue such as astrocytes, one could postulate that GFAP concentrations are elevated in the CSF of patients with SMA and decrease in response to nusinersen treatment, proposing cGFAP as a candidate biomarker in SMA.

In our study cohort, cGFAP concentrations in patients with SMA did not differ significantly from age‐ and sex‐matched controls before the start of treatment. In contrast, Olsson et al. reported higher cGFAP concentrations in children with SMA type 1 and 2 copies of *SMN2* gene (aged 0.5–4 months; controlled for age and sex).^23^ SMA type 1 is characterized by a fast disease progression and a rapid destruction of motor neurons. Our cohort mainly comprised of SMA type 2 and 3, which might be characterized by a less extended astroglial activation and subsequently lower cGFAP concentrations due to milder disease activity. Hence, the comparability of the two studies is limited.

Higher cGFAP concentration was associated with more impaired motor function and patients with SMA type 2 and non‐ambulatory patients had a higher cGFAP concentration than patients with SMA type 3 and patients who were still able to walk, respectively. This still supports the hypothesis of astrocyte involvement in SMA pathogenesis. However, despite the correlation of cGFAP concentration with disease severity, its limited applicability to distinguish SMA samples from control samples does not advocate for a diagnostic or prognostic use, at least in adult patients with SMA. For comparison of cGFAP with surrogate markers of neurodegeneration, we also determined cNfL concentrations, since these are known to reflect the extent of neurodegenerative processes in other motor neuron diseases, for example in amyotrophic lateral sclerosis. When investigating neurofilaments, one has to take into account their different subunits (e.g. phosphorylated neurofilament heavy chain (pNfH) versus NfL), the possibility of determination in different compartments (CSF versus blood) and their various detection methods (ELISA versus more sensitive methods as SIMOA and ELLA). As we reported before, pNfH in CSF was under lower limit of detection in most adult patients with SMA. In contrast, only few patients showed cNfL concentrations under the lower limit of detection measured with ELISA.^27^ Therefore, we decided to use the high sensitive method ELLA to measure cNfL as our cohort comprised primarily adult patients with SMA. In line with previous research, we found elevated cNfL concentrations in children with SMA type 1, but not in adult patients with SMA. We postulated that this might be due to the low number of remaining motor neurons as a consequence of advanced degeneration. However, we anticipated that the progressing degeneration of remaining motor neurons might be visible after normalization to the present motor neuron mass. For this purpose, we used sCrn as a surrogate measure for preserved muscle mass as it might reflect the amount of remaining motor units.^37^, ^38^ The results suggest that, although absolute values of cNfL in treatment‐naïve patients were similar to controls and did not differ between SMA type 2 and 3, patients with SMA type 2 had a relatively (sCrn‐normalized) higher release of cNfL implying a higher disease activity. The same was observed for cGFAP which matches the observation of higher cGFAP levels being associated with more impaired motor function. Longitudinally, cNfL decreased in the majority of our patients, which might demonstrate the beneficial effect of nusinersen treatment, particularly since cNfL has been found to increase during aging in healthy population.^39^

Furthermore, regarding SMA type 1, Olsson et al. reported decreasing cGFAP concentrations during nusinersen treatment in severely affected children with SMA and short treatment delay.^23^ Fitting to this observation, two of our patients with similar characteristics presented a remarkable decrease associated with a strong decline in cNfL and motoric improvement. Overall, however, cGFAP concentrations did not significantly change during nusinersen treatment in our cohort, which could be due to the diverse composition of our cohort and the very small proportion of patients with early disease onset and short treatment delay compared to Olsson et al. Still, two thirds of all patients showed a decline and the median cGFAP concentration after 14 months approximated the median cGFAP concentration of the control group. In addition, patients with improving HFMSE score during treatment presented a significant reduction of cGFAP concentrations. Since cGFAP was previously reported to increase with aging in healthy individuals,^6^, ^40^ we hypothesize that there might be an attenuation of astrogliosis during nusinersen treatment also in our cohort.

Our study has some limitations. Statistical analysis could be compromised by the small proportion of patients with SMA type 1 compared to type 2 and 3 within our study cohort. Also, GFAP was measured in the CSF compartment, which might not allow a conclusion about the origin of GFAP (upregulation of GFAP expression within the astrocytes during astroglial activation versus GFAP release in the context of astrocyte degeneration).

## Conclusion

GFAP concentration in CSF is not an outstanding biomarker in patients with long‐standing SMA, but might support the hypothesis that glial activation is involved in SMA pathology and may be modulated by nusinersen treatment. In contrast, NfL in CSF measured with high sensitive methods may be a promising biomarker in patients with SMA when normalized to preserved motor neuron mass, which should be subject to further studies along with a comprehensive investigation of the different neurofilament subunits in SMA.

## Conflict of Interest

MF reports non‐financial support from Biogen outside the submitted work.

PS reports no disclosures. CDW has received honoraria from Biogen as an advisory board member and for lectures and as a consultant and advisory board member from Hoffmann‐La Roche. She also received travel expenses from Biogen. OSK has received honoraria as a speaker/consultant and/or funding for travel expenses from the German Neuromuscular Society “Deutsche Gesellschaft fuer Muskelkranke” (DGM e.V.), Novartis, Biogen GmbH, Biermann Verlag GmbH, MK + S ‐ Medizin, Kommunikation & Service GmbH, and the Jain Foundation, outside the submitted work; and research support from the DGM e.V., outside the submitted work. AO has received honoraria from Biogen for lectures. SP has received grants from the German Neuromuscular Society, the Federal Ministry of Education and Research, the German Israeli Foundation for Scientific Research and Development, and the EU Joint Programme for Neurodegenerative Disease Research; and other support from Cytokinetics, Desitin Pharma, Biogen, Novartis, and Teva outside of the submitted work. JCK has received payment for consultation and advisory board participation from Biogen, Hoffmann‐La Roche and AveXis. KR has no conflicts of interest to declare that are relevant to the content of this article. AHu has no conflicts of interest to declare that are relevant to the content of this article. HT funding for research projects, lectures, and travel from Alexion, Bayer, Biogen, Celgene/Bristol‐Myers‐Squibb, GlaxoSmithKline, Janssen, Merck Serono, Novartis, Roche, Sanofi/Genzyme, Siemens and Teva, and received research support from Chemische Fabrik Karl Bucher GmbH, German Multiple Sclerosis Society (DMSG), and the German Ministry for Education and Research (BMBF). BW declares honoraria for lectures, travel support for meetings, and advisory board participation for Biogen, Novartis and Roche. BF has no conflicts of interest to declare that are relevant to the content of this article. ACL has received personal fees from AB Science, Biogen, Cytokinetics, GlaxoSmithKline, Orion Pharma, Novartis, Tau Rx Therapeutics, Teva, Mitsubishi, and Hoffmann‐La Roche outside of the submitted work. MO has no conflicts of interest to declare that are relevant to the content of this article. AH received royalties from BIOGEN and DESITIN as an advisory board member. RG has received honoraria from Biogen as an advisory board member and for lectures and as a consultant and advisory board member from Hofmann‐La Roche. He also received travel expenses and research support from Biogen.

## Authors' Contributions

All authors contributed to the acquisition of data and revised the manuscript for intellectual content. MF, PS, and RG analyzed and interpreted the data and prepared the original draft. AH and RG concepted and designed the study. RG did supervision and project administration.

## Consent for Publication

Not applicable.

## Ethics Approval

The local ethics committees of all participating sites approved the study and all patients and controls signed written informed consent.

## Supporting information


**Figure S1.** Correlation between cGFAP and cCHIT1 concentration before treatment initiation. Correlation (rho = 0.081, *p* = 0.483, *N* = 79) calculated by partial rank correlation controlling for patients' age and height. Shading distinguishes SMA type: light gray, SMA type 1; mid gray, SMA type 2; dark gray, SMA type 3. cGFAP, glial fibrillary acidic protein concentration in cerebrospinal fluid; cCHIT1, chitotriosidase 1 concentration in cerebrospinal fluid.
**Figure S2.** Individual dynamics of c GFAP concentrations during nusinersen treatment.
Individual longitudinal cGFAP raw data plotted against patients' age. Connected symbols represent the change of cGFAP concentration for an individual patient during 14 months of nusinersen treatment. *N* = 79
cGFAP, glial fibrillary acidic protein concentration in cerebrospinal fluid
**Figure S3.** Relationship between decreasing cGFAP and cNfL concentration and motor improvement. Individual data regarding cGFAP and cNfL concentration and motoric outcome of two selected patients (#74 and #57; both with disease onset and treatment initiation within the first year of life) who met the inclusion criteria by Olsson et al. (SMA type 1, 2 *SMN2* copies, treatment delay <4 years). Each upward tick on the x‐axis indicates the time of nusinersen administration. cGFAP (light green symbols) and cNfL (lilac symbols) measurement and CHOP INTEND assessment (black symbols) were done before treatment initiation (V1), after 6 months (V5) and after 14 months (V7) of nusinersen treatment. cGFAP, glial fibrillary acidic protein concentration in cerebrospinal fluid; cNfL, neurofilament light chain concentration in cerebrospinal fluid; *SMN2*, *Survival of motor neuron 2* gene; CHOP INTEND, Children's Hospital of Philadelphia Infant Test of Neuromuscular Disorders (higher score indicates better motor function).
**Figure S4.** Subgroup analysis of cGFAP and cNfL values normalized to sCrn. (A–C) Comparison of different variables between patients with SMA type 2 and 3 using Mann–Whitney *U* test (B) or one‐way analysis of covariance (ANCOVA) considering age as covariate (A and C). Boxes show interquartile range (IQR), whiskers indicate values within 1.5‐fold IQR, single icons show individual values outside the whisker range, bold horizontal line represents median, + indicates mean; color shading distinguishes SMA subtype. (D) Change of ratios during 14 months of nusinersen treatment. Colored circles represent median value of respected biomarker and subgroup; each tick on the x‐axis indicates a nusinersen administration. cGFAP, glial fibrillary acidic protein concentration in cerebrospinal fluid; cNfL, neurofilament light chain concentration in cerebrospinal fluid; sCrn, serum creatinine concentration; V1, baseline visit; V7, follow‐up visit after 14 months; *p < 0.05; **p < 0.01; ***p < 0.001; ****p < 0.0001.
**Table S1.** cNfL levels in treatment‐naïve patients with SMA (*N* = 73). cNfL, Concentration of neurofilament light chain in cerebrospinal fluid; IQR, interquartile range.
**Table S2.** Dynamics in cNfL concentration during 14 months of nusinersen treatment
cNfL, Concentration of neurofilament light chain in cerebrospinal fluid; IQR, interquartile range; Δ, difference vs baseline; *p* value calculated by Wilcoxon signed‐rank test, significant values are marked in bold.

## Data Availability

The datasets used and/or analyzed during the current study are available from the corresponding author on reasonable request.
