# Supplementary material for: Glial fibrillary acidic protein in cerebrospinal fluid of patients with spinal muscular atrophy
Source: Ann Clin Transl Neurol. 2022 Aug 11;9(9):1437–48. doi: 10.1002/acn3.51645 (PMC9463944; doi:10.1002/acn3.51645)
Supplement: Supplementary file 1 — Figure S1. Correlation between cGFAP and cCHIT1 concentration before treatment initiation. Correlation (rho = 0.081, p = 0.483, N = 79) calculated by partial rank correlation controlling for patients' age and height. Shading distinguishes SMA type: light gray, SMA type 1; mid gray, SMA type 2; dark gray, SMA type 3. cGFAP, glial fibrillary acidic protein concentration in cerebrospinal fluid; cCHIT1, chitotriosidase 1 concentration in cerebrospinal fluid. Figure S2. Individual dynamics of c GFAP concentrations during nusinersen treatment. Individual longitudinal cGFAP raw data plotted against patients' age. Connected symbols represent the change of cGFAP concentration for an individual patient during 14 months of nusinersen treatment. N = 79 cGFAP, glial fibrillary acidic protein concentration in cerebrospinal fluid Figure S3. Relationship between decreasing cGFAP and cNfL concentration and motor improvement. Individual data regarding cGFAP and cNfL concentration and motoric outcome of two selected patients (#74 and #57; both with disease onset and treatment initiation within the first year of life) who met the inclusion criteria by Olsson et al. (SMA type 1, 2 SMN2 copies, treatment delay <4 years). Each upward tick on the x‐axis indicates the time of nusinersen administration. cGFAP (light green symbols) and cNfL (lilac symbols) measurement and CHOP INTEND assessment (black symbols) were done before treatment initiation (V1), after 6 months (V5) and after 14 months (V7) of nusinersen treatment. cGFAP, glial fibrillary acidic protein concentration in cerebrospinal fluid; cNfL, neurofilament light chain concentration in cerebrospinal fluid; SMN2, Survival of motor neuron 2 gene; CHOP INTEND, Children's Hospital of Philadelphia Infant Test of Neuromuscular Disorders (higher score indicates better motor function). Figure S4. Subgroup analysis of cGFAP and cNfL values normalized to sCrn. (A–C) Comparison of different variables between patients with SMA type 2 and 3 usin [file ACN3-9-1437-s001.docx]

**Supplementary material**

**
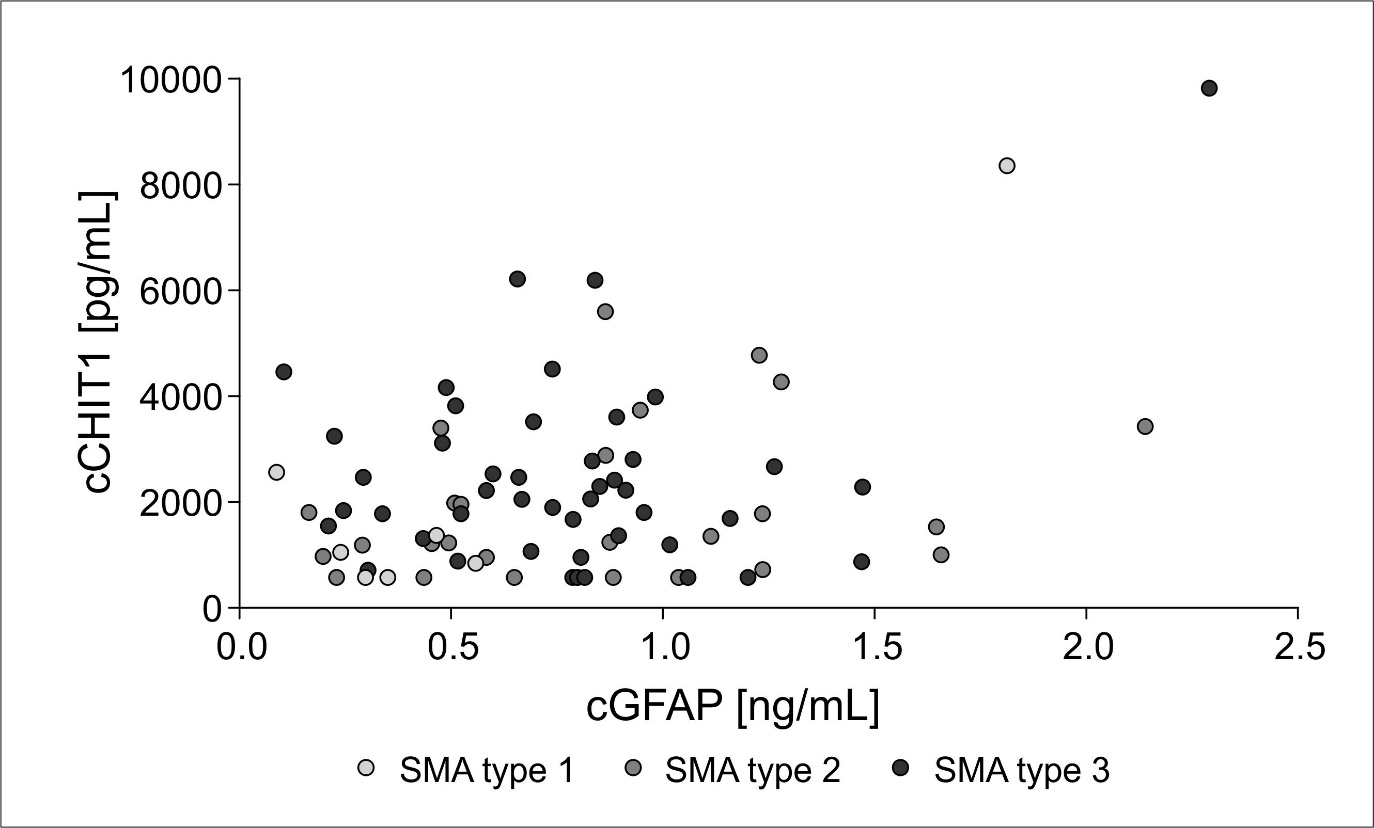
**

**Figure S1** Correlation between cGFAP and cCHIT1 concentration before treatment initiation.

Correlation (rho = 0.081, p = 0.483, N = 79) calculated by partial rank correlation controlling for patients’ age and height. Shading distinguishes SMA type: light gray, SMA type 1; mid gray, SMA type 2; dark gray, SMA type 3

cGFAP, glial fibrillary acidic protein concentration in cerebrospinal fluid; cCHIT1, chitotriosidase 1 concentration in cerebrospinal fluid

**
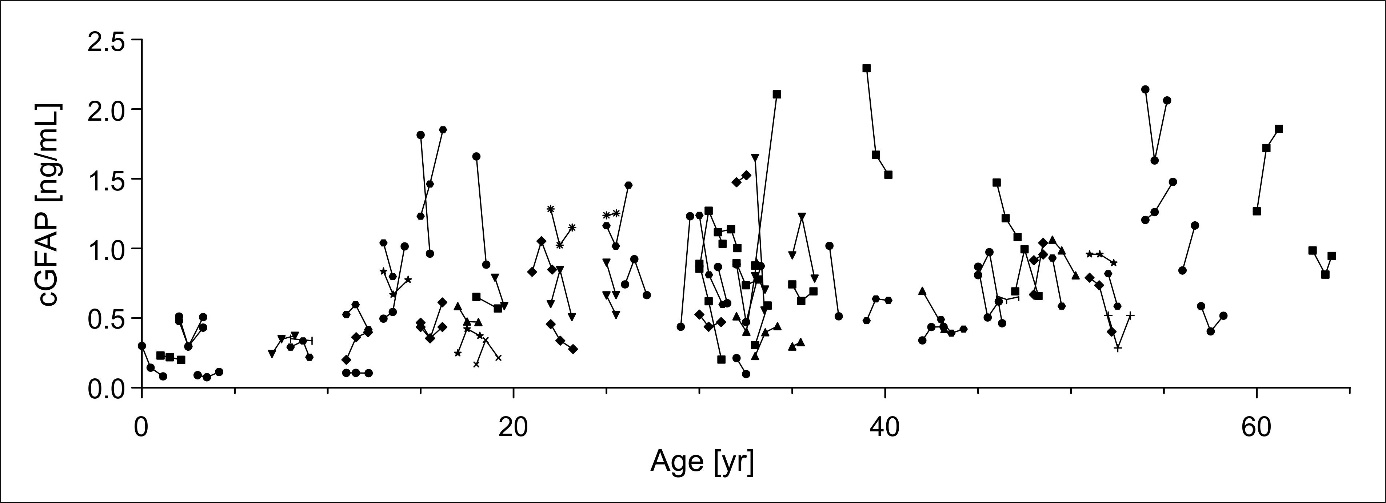
**

**Figure S2** Individual dynamics of c GFAP concentrations during nusinersen treatment.

Individual longitudinal cGFAP raw data plotted against patients’ age. Connected symbols represent the change of cGFAP concentration for an individual patient during 14 months of nusinersen treatment. N = 79

cGFAP, glial fibrillary acidic protein concentration in cerebrospinal fluid

**
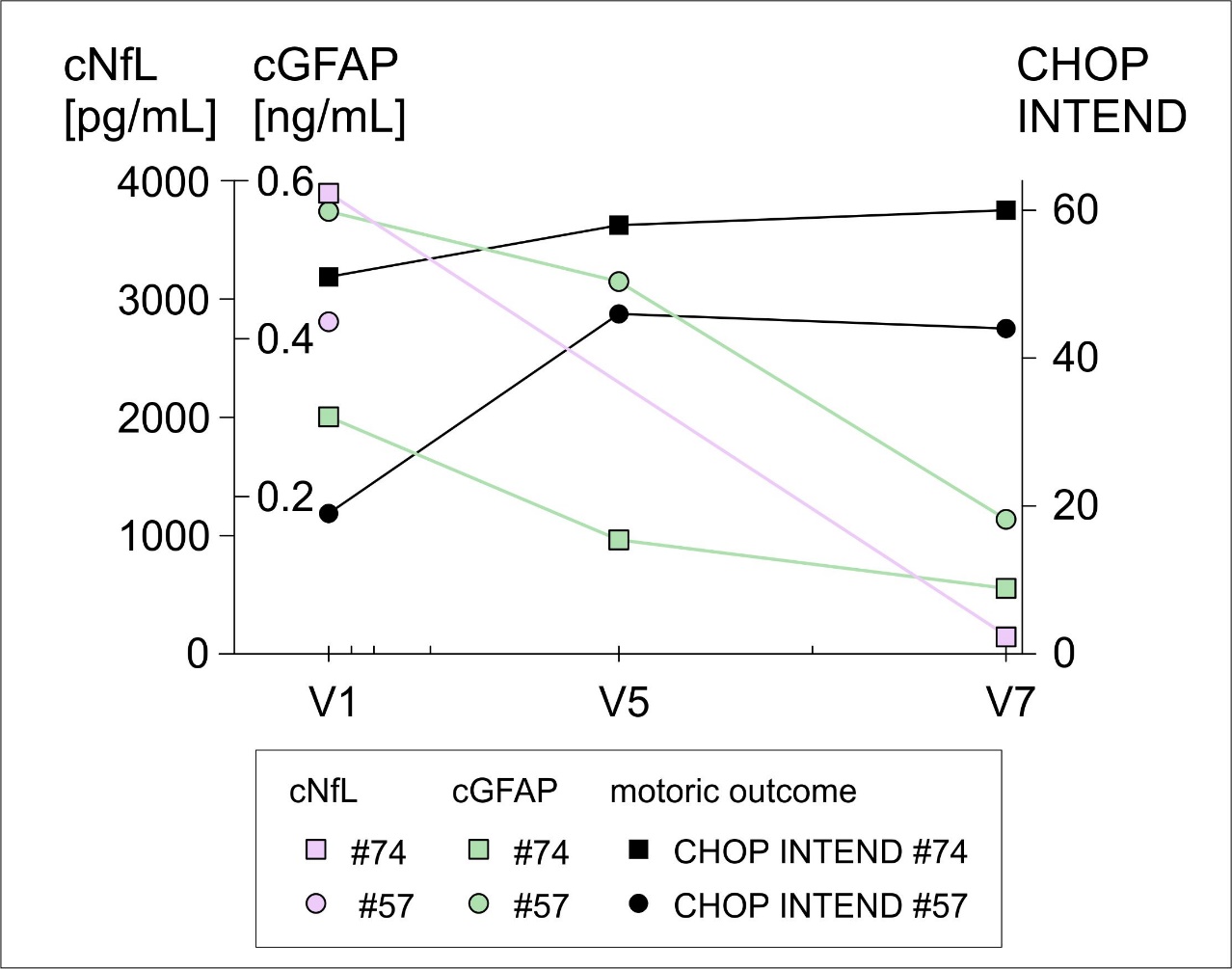
**

**Figure S3** Relationship between decreasing cGFAP and cNfL concentration and motor improvement.

Individual data regarding cGFAP and cNfL concentration and motoric outcome of two selected patients (#74 and #57; both with disease onset and treatment initiation within the first year of life) who met the inclusion criteria by Olsson et al. (SMA type 1, 2 *SMN2* copies, treatment delay < 4 years). Each upward tick on the x-axis indicates the time of nusinersen administration. cGFAP (light green symbols) and cNfL (lilac symbols) measurement and CHOP INTEND assessment (black symbols) were done before treatment initiation (V1), after 6 months (V5) and after 14 months (V7) of nusinersen treatment.

cGFAP, glial fibrillary acidic protein concentration in cerebrospinal fluid; cNfL, neurofilament light chain concentration in cerebrospinal fluid; *SMN2*, *Survival of motor neuron 2* gene; CHOP INTEND, Children’s Hospital of Philadelphia Infant Test of Neuromuscular Disorders (higher score indicates better motor function)

**
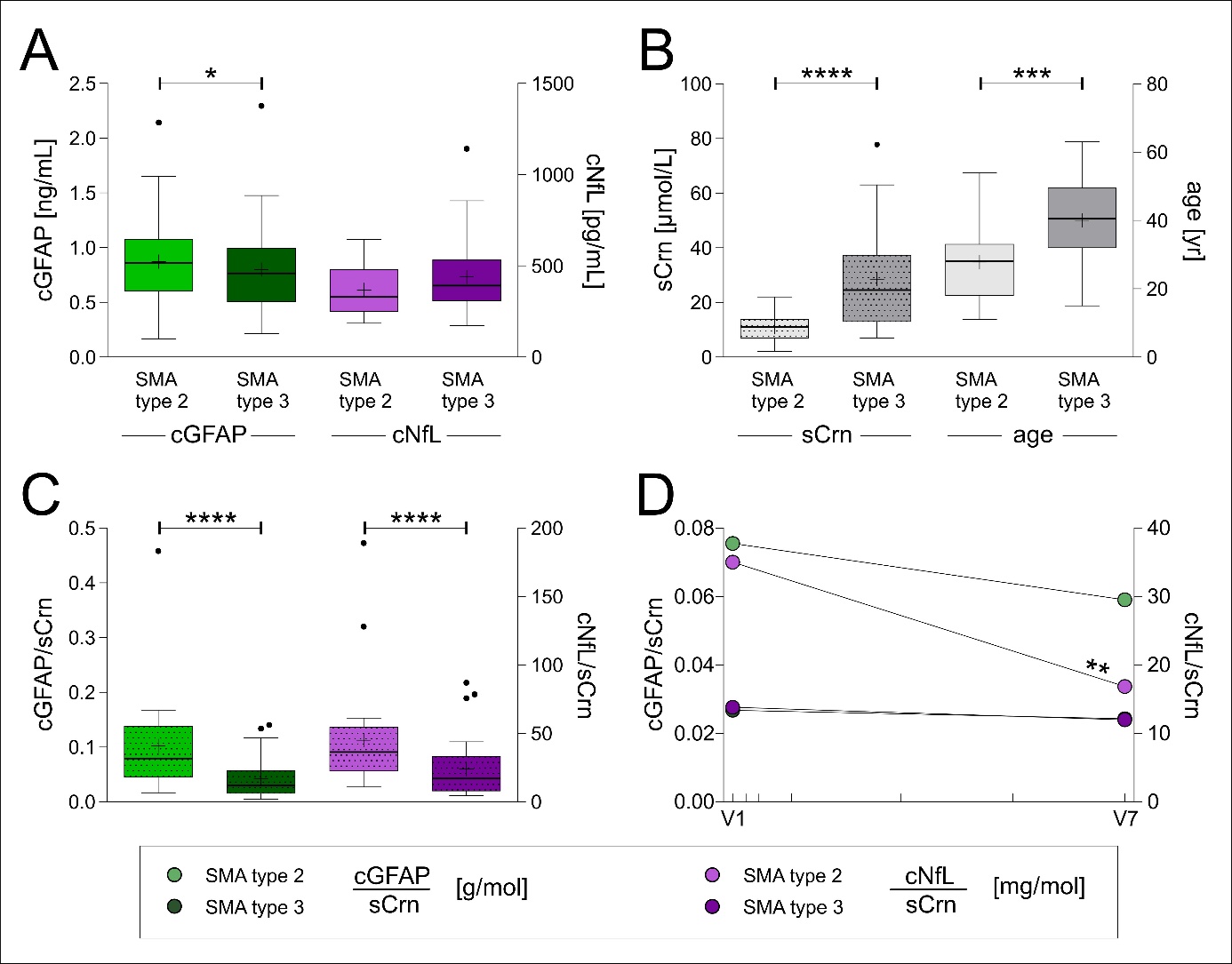
Figure S4** Subgroup analysis of cGFAP and cNfL values normalized to sCrn.

(A) – (C) Comparison of different variables between patients with SMA type 2 and 3 using Mann-Whitney U test (B) or one-way analysis of covariance (ANCOVA) considering age as covariate (A and C). Boxes show interquartile range (IQR), whiskers indicate values within 1.5-fold IQR, single icons show individual values outside the whisker range, bold horizontal line represents median, + indicates mean; color shading distinguishes SMA subtype.

(D) Change of ratios during 14 months of nusinersen treatment. Colored circles represent median value of respected biomarker and subgroup; each tick on the x-axis indicates a nusinersen administration.

cGFAP, glial fibrillary acidic protein concentration in cerebrospinal fluid; cNfL, neurofilament light chain concentration in cerebrospinal fluid; sCrn, serum creatinine concentration; V1, baseline visit; V7, follow-up visit after 14 months; *, p < 0.05; **, p < 0.01; ***, p < 0.001; ****, p < 0.0001

**Table S1** cNfL levels in treatment-naïve patients with SMA (N = 73)

|  | SMA type 1  (N = 5) | SMA type 2  (N = 31) | SMA type 3  (N = 37) |
| --- | --- | --- | --- |
| cNfL [pg/mL],  median  (IQR)  range | 993.0  (543.5 – 3344.0)  424 - 3885 | 320.0  (241.0 – 475.0)  159 - 645 | 389.0  (295.5 – 537.5)  173 - 1142 |

cNfL, Concentration of neurofilament light chain in cerebrospinal fluid; IQR, interquartile range

**Table S2** Dynamics in cNfL concentration during 14 months of nusinersen treatment

|  | Baseline | | 14-month analysis | |  |  |
| --- | --- | --- | --- | --- | --- | --- |
|  | N | median  (IQR) | median  (IQR) | Δ [%] | p value | |
| cNfL  [pg/mL] | 53 | 388.0  (295.5 – 505.5) | 236.0  (159.0 – 412.0) | -39 | **< 0.0001** | |
| SMA type 1 | 4 | 828.0  (483.75 – 3162.0) | 151.5  (106.7 – 182.5) | -82 | 0.068 | |
| SMA type 2 | 22 | 329.0  (236.5 – 485.5) | 185.0  (147.25 – 232.25) | -44 | **< 0.0001** | |
| SMA type 3 | 27 | 388.0  (302.0 – 498.0) | 373.0  (312.0 – 498.0) | -4 | 0.209 | |

cNfL, Concentration of neurofilament light chain in cerebrospinal fluid; IQR, interquartile range; Δ, difference vs baseline; p value calculated by Wilcoxon signed-rank test, significant values are marked in bold
